# A survey of new oncology drug approvals in the USA from 2010 to 2015: a focus on optimal dose and related postmarketing activities

**DOI:** 10.1007/s00280-015-2931-4

**Published:** 2016-01-25

**Authors:** Dan Lu, Tong Lu, Mark Stroh, Richard A. Graham, Priya Agarwal, Luna Musib, Chi-Chung Li, Bert L. Lum, Amita Joshi

**Affiliations:** Department of Clinical Pharmacology, Genentech, Inc., 1 DNA Way, MS 463A, South San Francisco, CA 94080 USA; Department of Clinical Pharmacology, Theravance Biopharma U.S., Inc., South San Francisco, CA USA

**Keywords:** Oncology, Dose selection, Pharmacokinetics, Modeling, Pharmacodynamics, Maximum tolerated dose, Food and Drug Administration

## Abstract

The maximally tolerated dose (MTD) of cytotoxic agents has historical precedence in treating cancer, as it was believed that dose and therapeutic effect are intrinsically linked and that the MTD would provide greatest therapeutic value. With molecularly targeted agents, the premise of preventing toxicity to normal tissues while modulating tumor growth provides a potential for an increased therapeutic window. Results from these targeted agents suggest we are entering an era of chronic cancer management, which will require design of regimens with long-term tolerability. A corresponding switch from MTD-based (toxicity-driven) dosing strategies to alternative paradigms is also expected. The challenge with these targeted agents is to fully understand the complex relationship between pharmacokinetics, pharmacodynamics, and safety and efficacy in early-stage trials, so that the optimal dose and schedule for registration trials may be identified. This review provides a systematic survey of the applications submitted to the United States Food and Drug Administration (FDA) for oncology indications, from 2010 through early 2015, and summarizes the dose selection rationale for registrational trials, the relationship of the MTD to outcomes of the final label dose, the postmarketing requirements or commitments related to dose optimization activities, the role of biomarkers, and typical exposure–response modeling methods.

**Electronic supplementary material:**

The online version of this article (doi:10.1007/s00280-015-2931-4) contains supplementary material, which is available to authorized users.

## Introduction: the changing environment for dose selection and optimization in oncology

A previous review of new drug applications submitted to the United States Food and Drug Administration (hereafter referred to as “FDA”) during 2000–2012 for different indications showed that drugs commonly failed to gain approval if they lacked a definitive optimal dose which maximizes efficacy and minimizes safety risks [1]. Parallel to this analysis, two recent publications on oncology drug development reviewed the challenges faced in selecting and optimizing doses for registration of clinical trials, and outlined potential strategies and data elements [2, 3]. With the continuing evolution of cancer drug development, a critical emerging issue is how to optimally dose agents for maximal efficacy, minimal toxicity, and optimal clinical application and cost-benefit [2–4].

Historically, dose selection of cytotoxic cancer chemotherapies considered the value of large and frequent doses which would provide significant tumor cell killing to achieve complete responses (fractional killing) and prevent development of resistance in residual clinically undetectable disease [5, 6]. Dose selection was usually done after treating small cohorts of patients, typically enrolled in Phase 1 dose escalation trials, who received a pre-specified dose in a cohort which resulted in clinically limiting toxicity (dose limiting toxicity; DLT). The “maximum tolerated dose” (MTD), a lower dose with more acceptable levels of toxicity, was then defined in a subsequent cohort. Therapeutic effect and tolerability were believed to be intrinsically linked, and the great risk–benefit considerations for cancers commonly dictated MTD selection for use in Phase 2 and registration trials.

Molecularly targeted agents hold promise to spare normal tissues from toxicity while acting upon specific receptors or factors that are critical to promote tumor survival and growth. A continuing increase in the number of reports of clinical success with these agents illustrate the potentially favorable therapeutic window for treatment with molecular targeted agents. These success stories suggest that we are entering an era where cancer is a disease to be managed chronically, making long-term tolerability of drugs and patient adherence increasingly important. As the transition from broadly cytotoxic to targeted therapies continues, there will be a corresponding switch from MTD-based dosing to an alternative paradigm that better resembles treatment of other chronic (non-oncologic) illnesses. Others have previously reviewed emerging concepts and strategies for determining the “optimal biologic dose” in an era of molecularly targeted and hormonal agents [2, 3]. These reviews highlight the current challenge we face in fully understanding the complex relationships between pharmacokinetics, pharmacodynamics, safety, and efficacy in early-stage trials, where we aim to define the optimal dose for registration trials for oncology drug development.

Recent reviews have proposed that certain clinical trial design strategies and necessary data elements are required to enable identification of the optimal dose across all phases of clinical development. These elements include trial design features such as adaptive designs or randomized dose explorations, coupled with adequate assessment of pharmacokinetics and pharmacodynamics (i.e., biomarker response and clinical effect) in early clinical trials, to assess relationships between drug exposure, target effect, and acute toxicity [2, 3]. In later phases of development, it is also important to study the relationship of drug exposure to long-term clinical outcomes (both safety and efficacy) in the target patient population, where sparse pharmacokinetic sampling in registration trials would provide valuable insights on the optimal dose. Another important attribute of these strategies is the study of patient characteristics that determine pharmacokinetic variability, such as demographics (i.e., body weight, age, sex, etc.), physiologic factors (e.g., organ function), pathophysiological conditions related to tumor/disease status, pharmacogenomics, co-medications, and others, which allow for further dose optimization for specific patient populations.

A previous review presented seven examples of recently approved oncology products to demonstrate that alternative doses are being evaluated in postmarketing trials, indicating that the label dose may not be the optimal dose [3]. To extend the knowledge of dose selection and optimization during clinical development, we conducted a comprehensive survey of the label dose for New Molecular Entity (NME) applications for oncology drugs approved by the FDA from 2010 through the first quarter of 2015. This relatively narrow time frame was arbitrarily selected on our belief that exposure–response analyses may have been more consistently applied in the recent years of the drug review and approval process.

The key objectives of this survey are to systematically:

1. Evaluate the frequency of dose optimization-related postmarketing requirements (PMR)/postmarketing commitments (PMC);
2. Assess the outcomes for use for the MTD as the recommended dose for registration trials and the outcome of this dose selection strategy;
3. Summarize the use of upwards dose titration as a strategy of dose optimization;
4. Provide representative examples of biomarkers as a guide to dose selection/justification,
5. Present an overview of the pharmacometric methods of exposure–response (E–R) analysis in NME applications.

## Data sources

 This survey included NME New Drug Applications (NDA) or Biologics License Applications (BLA) for oncology indications that were approved by the FDA between 2010 and through the first quarter of 2015. The information reviewed includes the “clinical pharmacology and biopharmaceutics review” documents publicly available from http://www.accessdata.fda.gov/scripts/cder/drugsatfda/. The primary focus was on the exposure–response (E–R) summary, relating to the following question by the FDA reviewer: “*is the dose and dosing regimen selected by the applicant consistent with the known relationship between dose–concentration–response?*”. Label and approval letters were reviewed to summarize the current label dose and the PMRs/PMCs [7] related to the label dose justification or optimization. Selected publications related to label dose justification for these drugs were also reviewed. The term “dose justification” is used to reflect the fact that the proposed label dose in the application is supported by clinical data based on FDA review, while “dose optimization” means that the sponsor was asked to consider post-approval activities such as PMR or PMC to identify any alternative dose which confers a better risk–benefit profile.

PMR and PMC refer to studies and clinical trials that sponsors conduct after FDA approval, to gather additional information about a product’s safety, efficacy, or optimal use. Further definition of PMRs includes studies and clinical trials that sponsors are required to conduct under one or more statutes or regulations. PMCs are studies or clinical trials that a sponsor has agreed to conduct, but that are not required by a statute or regulation [7, 8].

## Dose optimization-related PMR/PMC for an oncology NME are common

**Fig. 1:**
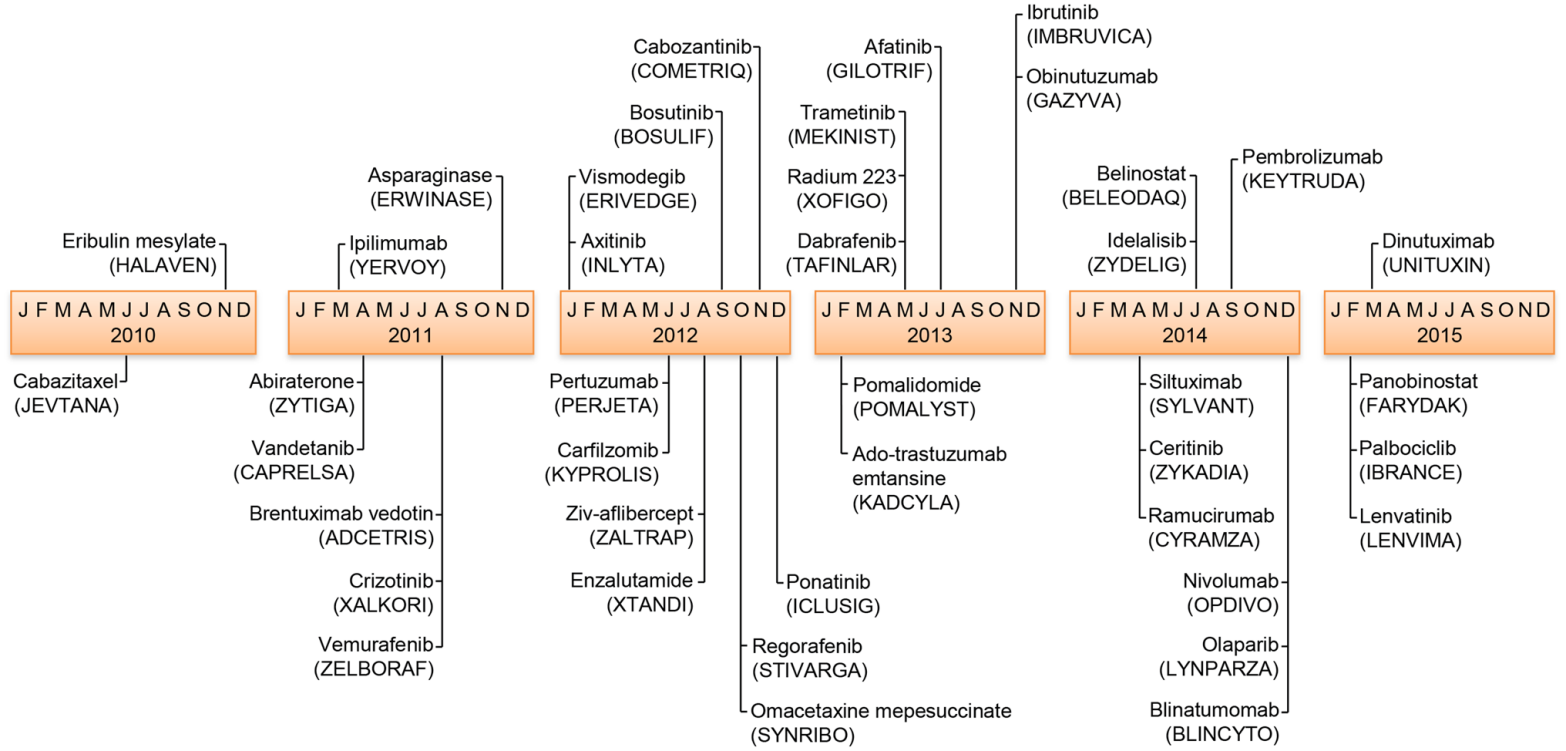
Chronological order of 41 NMEs approved by the FDA to treat cancer indications during the survey period 2010 through the first quarter of 2015

During the survey period, the FDA approved 41 NME applications through the NDA or BLA review processes for cancer indications. Figure 1 provides chronological depiction of these drugs and their approval, which includes 13 large molecules (LM) and 28 small molecules (SM). Subclassifications of the LMs resulted in nine monoclonal antibodies (mAbs), two antibody drug conjugates (ADCs), one enzyme, and one fusion protein. Subclassifications of the SMs resulted in 16 kinase inhibitors (KIs), eight non-KI small molecule-targeted agents, three chemotherapeutic agents, and one radioactive agent (Fig. 1, Table 1, Supplemental Table 1).

**Table 1:**
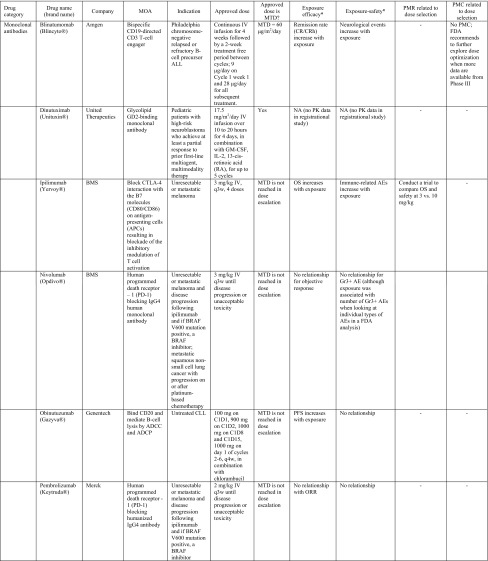

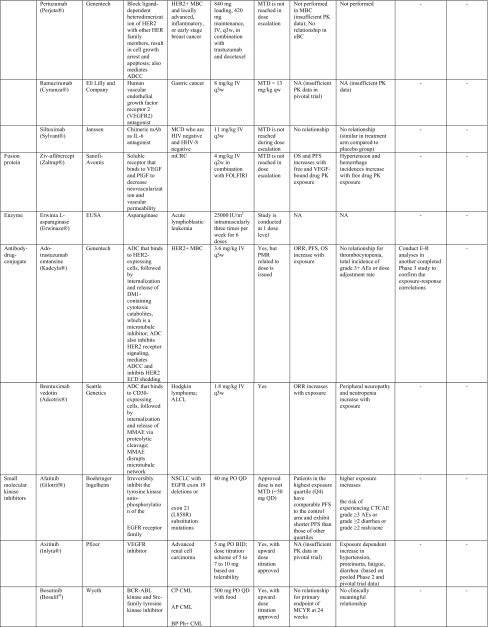

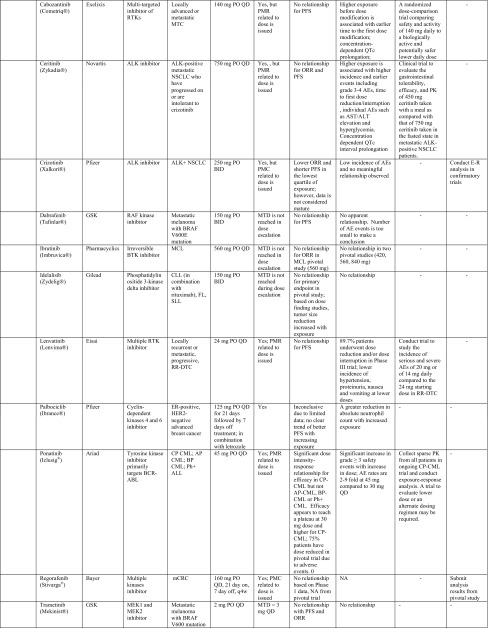

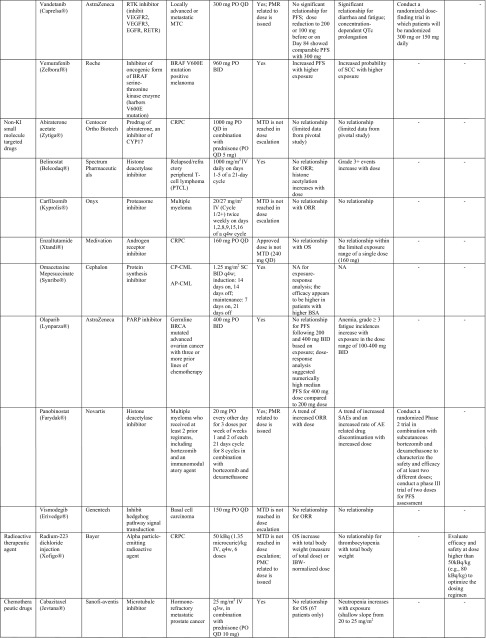

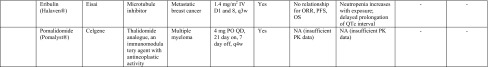
Summary of label dose justification and post-marketing requirement/commitment for dose optimization of oncology drugs (*n* = 41) approved by the US Food and Drug Administration from 2010 to Q1 2015

*ADC* antibody drug conjugate, *ADCC* antibody-dependent cell-mediated cytotoxicity, *ADCP* antibody-dependent cellular phagocytosis, *AEs* adverse events, *ALCL* anaplastic large cell lymphoma, *ALK* anaplastic lymphoma kinase, *ALL* acute lymphoblastic leukemia, *AP*-*CML* accelerated phase chronic myelogenous leukemia, *BCR*-*ABL* break point cluster-Abelson tyrosine kinase fusion protein gene, *BID* twice per day, *BP*-*Ph* + *CML* blast phase Philadelphia chromosome-positive chronic myelogenous leukemia, *BRAF* a human gene that makes a protein called B-Raf, a member of the Raf kinase family, *BSA* body surface area, *BTK* Bruton’s tyrosine kinase, *C1D1* cycle 1, day 1, *CLL* chronic lymphocytic leukemia, *CML* chronic myeloid leukemia, *CP*-*CML* chronic phase chronic myelogenous leukemia, *CRC* colorectal cancer, *CRPC* castration-resistant prostate cancer, *CTCAE* common terminology criteria for adverse events, *CTLA*-*4* cytotoxic T-lymphocyte antigen 4, *CYP17* 17α-hydroxylase/C17,20-lyase; a key enzyme in the production of androgens in the testes and adrenal glands, *eBC* early breast cancer, *ECD* extracellular domain, *EGFR* epidermal growth factor receptor, *E–R analyses* exposure–response analyses, *FL* follicular B cell non-Hodgkin Lymphoma, *FOLFIRI* 5-fluorouracil, leucovorin, irinotecan, *GM*-*CSF* granulocyte–macrophage colony-stimulating factor, *GSK* GlaxoSmithKline, *HER2* human epidermal growth factor receptor 2, *HHV*-*8* human herpesvirus-8, *HIV* human immunodeficiency virus, *IL*-*2* interleukin-2, *IBW* ideal body weight, *IV* intravenous, *MBC* metastatic breast cancer, *MCD* multicentric Castleman’s disease, *MCL* mantle cell lymphoma, *mCRC* metastatic colorectal cancer, *MCYR* major cytogenetic response, *MEK* mitogen-activated extracellular signal regulated kinase, *MMAE* monomethyl auristatin E, *MOA* mechanism of action, *MTC* medullary thyroid cancer, *MTD* maximum tolerated dose, *NA* not assessed, *NSCLC* non-small cell lung cancer, *ORR* objective response rate, *OS* overall survival, *PARP* poly ADP ribose polymerase, *PD*-*1* human programmed death receptor—1, *PFS* progression-free survival, *PlGF* placenta growth factor, *PK* pharmacokinetic(s), *PMC* postmarketing commitment, *PMR* postmarketing requirement, *QTc* corrected QT interval, *q3w* every-three-weeks, *PO* per os, *QD* one-a-day, *QID* 4 times per day, *RA* retinoic acid, *RAF* Raf kinase family, *RCC* renal cell carcinoma, *RETR* RET receptor tyrosine kinase, *RTK* receptor tyrosine kinases, *SC* subcutaneous injection, *SCC* squamous cell carcinoma, *SLL* small lymphocytic lymphoma, *TKIs* tyrosine kinase inhibitors, *VEGF* vascular endothelial growth factor, *VEGFR* vascular endothelial growth factor receptors

* For exposure–efficacy and exposure–safety analysis, when no relationship is indicated, it refers to that observed within the exposure range used in the analysis given the doses studied (for example, pivotal study only or pooled analysis of several studies), there does not appear to be a trend of relationship. This may be inconclusive as most pivotal trials only test one dose level that did not result in a wide range of exposures. It is likely that a relationship exists but the lower exposures needed to reveal this relationship, were not studied in the analysis

Eleven of the 41 (26.8 %) NME approvals had dose optimization-related PMR/PMCs issued by the FDA, based on clinical reviews and E–R analysis results performed by sponsors and/or FDA reviewers (Table 1). Overall, the tendency to receive a PMR/PMC for dose optimization activities appears slightly higher for TKIs than other drug classes.

PMR/PMC dose optimization activities include requests for conducting additional clinical studies and/or E–R analyses for dose optimization/identification. Seven drugs received PMRs/PMCs to conduct clinical studies for evaluation of whether an alternative to the proposed label dose would have a better benefit–risk profile. These included two evaluations of whether a higher dose would improve efficacy-1 mAb (ipilimumab) [9] and 1 radioactive agent (radium-223) [10] and five instances of whether a lower dose would provide a better safety profile without compromising efficacy, including the small molecule KI and non-KI agents (vandetanib, cabozantinib, ceritinib, lenvatinib, and panobinostat) [11–19]. Four other drug approvals resulted in PMRs/PMCs for additional E–R analysis results to support the proposed label dose, including one ADC (ado-trastuzumab emtansine) [20] and three KIs (ponatinib, regorafenib, and crizotinib) [21–24]. Further details on the label dose, correlation with MTD, E–R analysis results (in support of the label dose), and PMRs/PMCs for dose justification/optimization issued for these 41 drugs are provided in Table 1.

An overview of pharmacometric methods for E–R analysis in NME applications is provided later in this review.

## Approval outcomes of label dose related to MTD

**Fig. 2:**
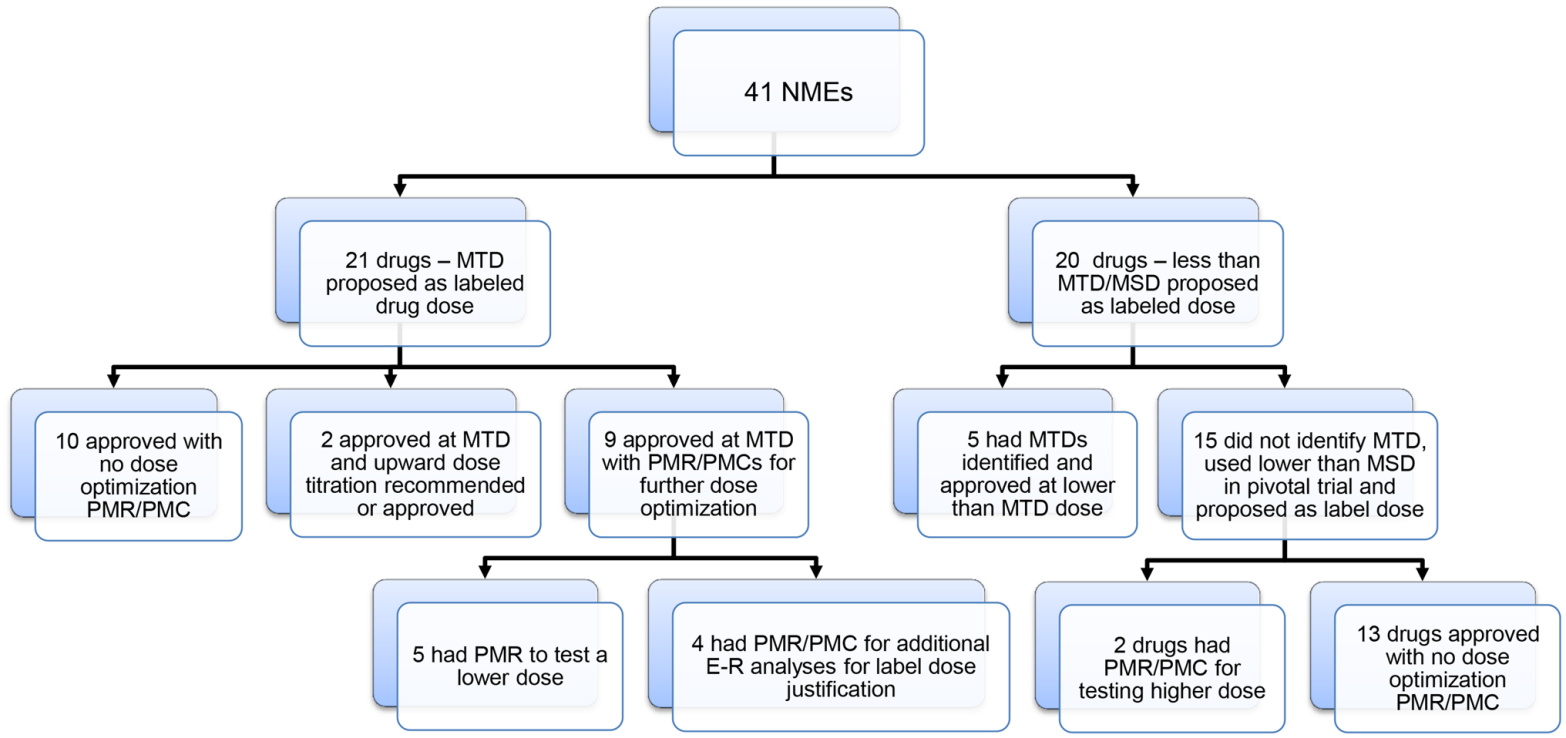
Summary of dosing paradigms for the 41 NMEs approved to treat cancer from 2010 through the first quarter of 2015

Figure 2 illustrates outcomes for the 41 drugs surveyed, with or without a development strategy at the MTD. For 26 of the 41 (63.4 %) NMEs, the MTD was identified during dose escalation trials, and a MTD was not reached for the remaining 15 drugs. For the 26 drugs with an identified MTD, 21 (80.8 %) instances, the MTD was selected for the registration trials and also proposed by the sponsor as label dose, suggesting that the MTD dosing paradigm dominates in oncology drug development. As shown in Fig. 2, for 21 of the 41 (51.2 %) NMEs, the MTD was proposed as the label dose and for the remaining 20 of the 41 (48.8 %) NMEs, a dose lower than the MTD or maximum studied dose (MSD) was proposed as the label dose. Among the 21 drugs with the proposed MTD as the initial starting dose, this labeling was found to be justified following FDA review in only 47.6 % (10 out of 21) instances, using criteria of no PMR/PMC for further dose optimization or no upwards dose titration recommendations (Fig. 2, Table 1). These 10 drugs include a variety of therapeutic classes, including one mAb (dinutuximab), three chemotherapeutic drugs (cabazitaxel, eribulin, and pomalidomide), two KI (vemurafenib and palbociclib), three non-KI targeted agents (omacetaxine mepesuccinate, olaparib, and belinostat) and one ADC (brentuximab vedotin). Two drugs (bosutinib and axitinib), where the approved label dose was the MTD, had an upward dose titration strategy to achieve dose levels above the MTD for individual patients meeting certain criteria, either recommended by the sponsor (axitinib) or FDA (bosutinib). Nine of 21 (42.9 %) of drugs developed at the MTD had dose optimization PMRs/PMCs issued by the FDA. In five instances (vandetanib, cabozantinib, ceritinib, lenvatinib, and panobinostat) PMR clinical trials were requested to test a lower dose for safety concerns and in four instances (ponatinib, regorafenib, crizotinib, and ado-trastuzumab emtansine) additional E–R data were to be provided for further label dose justification.

Among 20 drugs where the sponsor proposed a label dose lower than the MTD/MSD, five (25 %) had a MTD identified but proceeded with clinical development and registration trial using lower doses (afatinib, trametinib, enzalutamide, ramucirumab, and blinatumomab). In all five cases, the proposed label dose was justified and no dose optimization postmarketing activities were issued (Fig. 2, Table 1). For the remaining 15 drugs where the MTD was not identified in early dose escalation trials, sponsors were able to successfully justify the dose used in the registration trial and the proposed label dose in 13 out of 15 (86.7 %) instances, typically by using exposure–efficacy/exposure–safety (E–E/E–S) analysis. For the remaining two cases (ipilimumab and radium-223), PMRs were issued to test whether a higher dose would provide a greater degree of clinical benefit.

The mAbs are the largest category among large molecules approved with nine approvals in the past 5 years. For six of these mAbs (ipilimumab, nivolumab, obinutuzumab, pembrolizumab, pertuzumab, and siltuximab), a MTD was not reached/identified during Phase 1 dose escalation studies, and a dose lower than the MSD was proposed as the label dose (Table 1). Of these, ipilimumab received a PMR to assess efficacy and safety for a higher dose (10 mg/kg), as compared to the approved label dose of 3 mg/kg [25, 26]. For the remaining three mAbs (blinatumomab, dinutuximab, and ramucirumab), the MTD was determined during dose escalation. For dinutuximab, the MTD is proposed as the label dose; for blinatumomab and ramucirumab, the proposed label dose was lower than the MTD with none of the three drugs receiving dose optimization-related PMR/PMCs. It is worth noting that blinatumomab is a novel type of mAb which is a bi-specific CD19-directed CD3 T cell engager.

Two ADCs were approved during the survey period, brentuximab vedotin and ado-trastuzumab emtansine [20, 27]. In both cases, MTDs were determined in early clinical trials and the MTD was selected for registration trials. For brentuximab vedotin, the FDA review concluded that the label dose balanced efficacy and safety well, and no PMR/PMC activities were suggested. For ado-trastuzumab emtansine, the FDA review suggested that higher drug concentration correlated with better efficacy measures, though there was no apparent concentration relationship to safety. It was concluded that there may be an opportunity to improve clinical benefit by optimizing (increasing) the dose for low-exposure patients. A PMC analysis was implemented by the sponsor to further characterize the E–R relationship using data from a Phase 3 trial that was ongoing during this review, to inform if a new dose optimization trial should be performed for a potential dose optimization clinical trial [20, 28–32].

**Fig. 3:**
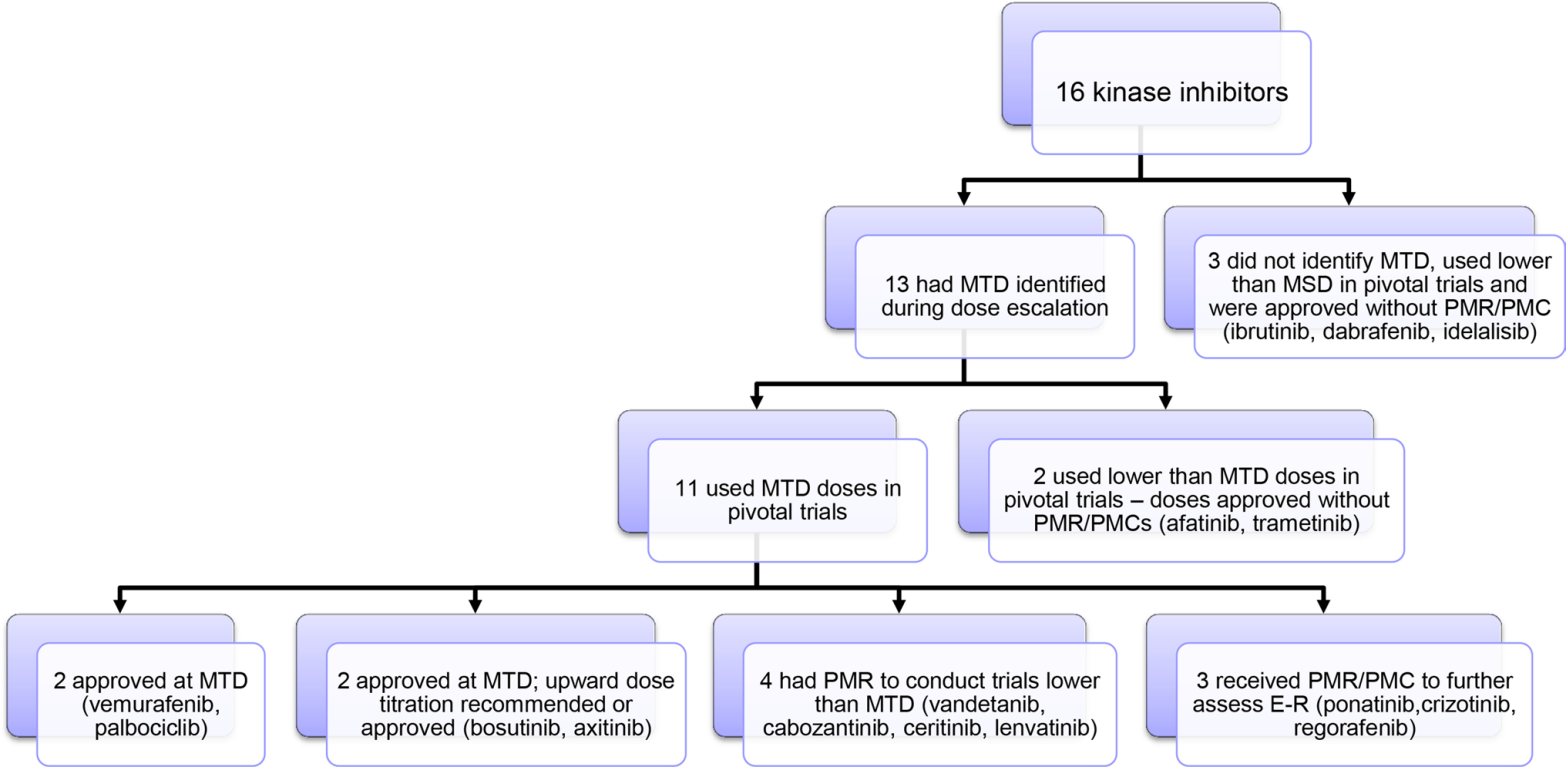
Summary of dosing paradigms for small molecule TKIs

Kinase inhibitors were the largest category within the SM classification (16 out of 28 SMs). During the survey period, 16 KIs were approved and accounted for 39.0 % of the 41 approvals. Figure 3 summarizes the dosing paradigm for these 16 KIs and their drug approval outcomes. Thirteen of the 16 (81.3 %) KIs had their MTD identified during early clinical dose escalation trials (Fig. 3). Eleven of these 13 drugs (84.6 %) had the MTD proposed as the label dose for registration trials, again suggesting that the MTD dosing paradigm dominates for KIs. Upon review of the NDA, the FDA challenged the MTD as the label dose in most cases, with vemurafenib and palbociclib representing the only approvals of the MTD as the label dose [33] (Table 1). In the cases of bosutinib and axitinib, although the MTD was approved as the label dose, upward dose titration was approved. For bosutinib, dose titration was not used in the pivotal study; however, FDA recommended upward dose titration for bosutinib based on individual patient efficacy and tolerability, based on a retrospective analysis. For axitinib, upward dose titration was tested in the pivotal study based on individual patient safety/tolerability, and was approved by the FDA based on PK and exposure–response analysis [34, 35]. For the remaining 7 KI drugs with approval of a label dose at the MTD, vandetanib, cabozantinib, ceritinib, and lenvatinib received PMRs to conduct trials testing lower doses for safety concerns [11–14] and 3 others received PMRs/PMCs to further assess E–R relationships using data in the ongoing Phase 3 trial (ponatinib and crizotinib) [21, 23] or to submit a complete package of E–R assessment (regorafenib) [22] (Table 1). Among the 13 drugs with a MTD identified, the sponsors proposed registration trials at a lower dose than the MTD for two drugs (afatinib and trametinib) and both doses were approved with no postmarketing optimization requirements [36, 37]. Out of 16 KIs that did not have an MTD identified and underwent development at a dose lower than the MSD, three drugs (ibrutinib, dabrafenib, and idelalisib) were approved by the FDA with no PMR/PMC dose optimization requirements being issued (Fig. 3 and Table 1) [28, 38, 39].

For eight non-KI small molecule-targeted drugs approved during the survey period, MTD was reached in five drugs and was proposed as the label dose in four of them, except for enzalutamide, for which a lower than MTD dose was used in the registration trial and approved as the label dose [15, 40–43] (Table 1). For these five drugs, four received approval of the proposed label dose without PMR/PMCs (belinostat, omacetaxine, olaparib, and enzalutamide). For panobinostat, a PMR was issued to conduct a randomized Phase 2 trial to characterize the safety and efficacy of at least two different doses and to conduct a Phase 3 trial of two doses for progression-free survival (PFS) assessment. For the three non-KI small molecule-targeted drugs (abiraterone acetate, carfilzomib, and vismodegib) [44–46] where MTDs were not reached during dose escalation trials, doses lower than the MSDs were proposed for study in the registration trials and emerged as the label doses and no dose optimization-related PMR/PMC was issued for these three drugs [44–46].

Three chemotherapeutic drugs (cabazitaxel, eribulin, and pomalidomide) used MTDs for study in the registration trials which were approved with no PMRs/PMCs related to dose optimization issued to the sponsors [47–49].

For the radioactive agent radium-223 dichloride injection, no MTD was determined in the Phase 1 dose escalation trial and the label dose was proposed to be 50 kBq/kg. After review, FDA analysis suggested that patients with lower body weight (BW) had poorer overall survival (OS), while the incidence of Grade 3 or higher adverse events (AEs) was similar across a BW range. These findings resulted in a PMR issued [10] to conduct a randomized Phase 2 trial to evaluate the efficacy and safety of a dose higher than 50 kBq/kg [50].

Overall, the survey data suggest that while the development of oncology drugs at the MTD is still common (51.2 % of 41 cases and 80 % when the MTD is previously identified), success of the MTD as a justified label dose is infrequent, with nearly one-half of these NDA/BLA applications resulting in PMRs/PMCs issued for further dose optimization activities. PMR/PMC dose optimization activities occurred across all of the drug classes. The strategy for the development of novel KIs during the survey period appears to follow the historical MTD development paradigm, with the majority entering clinical development with doses selected for registration trials and proposed label doses at the MTD (11 out of 16 KIs); however, despite their approval, only two out of 11 drugs had label doses equivalent to the proposed MTD and seven out of 11 drugs had PMRs/PMCs issued for dose optimization efforts due to safety concerns. This suggests that the current dosing paradigm for KIs needs to be shifted.

## Upwards dose titration as a strategy of dose optimization

Most drugs have a specified downward dose titration method to manage AEs in their labels. Upward dose titration is an approach to individualize and potentially optimize doses to reduce inter-subject PK variability, thus balancing efficacy and toxicity and increasing clinical benefit. Two (7.1 %) of the 28 approved NMEs (afatinib and axitinib) tested an upward dose titration approach in their clinical development with axitinib approved for this approach while afatinib not approved [34–36, 51]. FDA approved bosutinib label with upward dose titration recommendations, although this was not tested in the pivotal study.

For axitinib, the approved dosing strategy includes upward dose titration based on patient tolerability. The MTD established for axitinib in early clinical trials was 5 mg twice daily without food. Analysis of three phase 2 trials showed that a higher value for the area under the curve (AUC) was associated with longer PFS and OS and a higher overall response rate (ORR) in renal cell carcinoma [35, 52]. However, greater exposure also correlated with a higher probability of toxicity, thus guiding the sponsor to implement a dose titration scheme in their Phase 2 and Phase 3 registration trials. This dosing scheme comprised of a starting dose of 5 mg twice daily, increased up to a maximum of 10 mg twice daily, based on tolerability. Retrospective analysis showed that there was considerable inter-subject variability in pharmacokinetics before dose titration and that patients who were able to tolerate axitinib upward dose titration had lower initial exposures. In these subjects, axitinib pharmacokinetics after dose titration appeared to match with those observed in subjects with no dose titration [35]. Clinical efficacy in patients with or without dose titration was further compared in a blinded Phase 2 trial; dose titration was found to improve the ORR [53]. Dose reductions to manage hypertension and proteinuria were also justified. Based on the above E–E and E–S analysis, as well as the positive registration study, which suggested an overall clinical benefit, the upward dose titration and dose reduction schemes were approved by the FDA [35]. The success of the axitinib dose titration strategy is facilitated by the ease of routine noninvasive assessment of the endpoints monitored for titration by the clinician and patient, e.g. blood pressure, fatigue, proteinuria, and diarrhea.

Afatinib is an example where the strategy of an upward dose titration did not result in clinical benefit. The registration trial used a dose of 40 mg, which was lower than the MTD of 50 mg, and was believed to be better tolerated. The design of the registration trial allowed for an upward dose escalation to 50 mg based on individual tolerability. E–R analysis of the trial indicated that patients in the highest exposure quartile had shorter PFS than those of other quartiles and that the higher exposure also increased the risk of AEs. Clinical observations also showed that 10 out of 16 patients (63 %) who were escalated to the 50 mg daily dose subsequently experienced dose reduction to 40 or 30 mg. The results of the E–R analysis and the high percentage of dose modification led to the FDA recommendation for capping the daily dose of afatinib to a maximum of 40 mg [36].

For bosutinib, a BCR-ABL kinase and Src-family KI indicated in CML, the registration trial was divided into two parts: Three doses were tested in Part I of the study, and the 500 mg dose with food was selected as the MTD for the Part II efficacy evaluation. While the bosutinib label dose is 500 mg [54], the FDA provided the following recommendation for upward dose titration; “consider dose escalation to 600 mg once daily with food in patients who do not reach complete hematological response by week 8 or a complete cytogenetic response by week 12, who did not have Grade 3 or higher adverse reactions, and who are currently taking 500 mg daily.” The rationale for this statement was not outlined in the “clinical pharmacology and biopharmaceutics reviews” for bosutinib [34], but presumably is intended to maximize the potential for benefit in patients adequately tolerating bosutinib.

## Biomarkers as a guide to dose selection/justification

An exposure–biomarker relationship was provided by sponsors to justify the label doses for 5 NMEs: ibrutinib, trametinib, enzalutamide, abiraterone acetate, and carfilzomib [37, 38, 41, 44, 45] (Table 1).

Ibrutinib, a drug targeting the Bruton’s tyrosine kinase (BTK) receptor, was approved to treat mantle cell lymphoma. No MTD for ibrutinib was established during dose escalation in early clinical trials, and a dose of 560 mg/day was proposed for the registration trial and approved as label dose. Dose selection of this drug was based on receptor occupancy, which had been shown to correlate with clinical efficacy. In the Phase 1 trial in which doses ranging from 1.25 to 12.5 mg/kg were studied, a maximum BTK receptor occupancy of >90 % and highest ORR was achieved at doses >2.5 mg/kg (>175 mg/day for 70 kg subject). The dose of 560 mg/day selected for the registration trial is therefore approximately threefold higher than the dose which provided a high degree of target binding, prevented lower exposure as a result of PK variability, demonstrated acceptable toxicity, and clinical activity in the early trial. FDA review summary documents note that a lower dose could be considered in future development; however, clinical benefit was established at a 560 mg/day label dose and no PMR/PMCs were issued for dose optimization [38].

Trametinib is a drug which inhibits the MEK pathway and was developed to treat metastatic melanoma with a BRAF V600 mutation. While an MTD of 3 mg was determined in a dose escalation trial, development proceeded at a dose of 2 mg in Phase 2 and 3 trials [37]. Dose selection appeared to be based on the following rationale: (1) the long-term tolerability at the MTD of 3 mg was of concern with chronic treatment in the Phase 1 trial; there were fewer AEs at 2 mg versus 2.5 and 3 mg doses, (2) the mean concentration at the 2 mg once daily dose exceeded the preclinical target, (3) the response rate was similar at 2 and 2.5 mg based on data from the Phase 1 trial, and (4) inhibition of pERK and Ki67, and enhancement of p27 in paired tumor biopsy specimens before and after treatment showed that trametinib at the 2 mg dose was more effective in modulating the biomarkers than at 0.5 and 1 mg [55]. In Phase 2 and registration trials, only the 2 mg dose was studied, and no clear E–R relationships were found. The FDA approved 2 mg as the label dose based on the multiple supportive factors listed above [37].

For enzalutamide, an androgen receptor inhibitor for treating castration-resistant prostate cancer, early clinical trials established the MTD to be 240 mg/day, yet the 160 mg/day dose was studied in the registration trial, which resulted in the approved label dose. The dose selection of 160 mg was supported by the PSA response in the Phase 1 trial.

Abiraterone acetate is an androgen biosynthesis inhibitor that works by inhibiting the 17α-hydroxylase/C17,20-lyase (CYP17) pathway and was developed to treat castration-resistant prostate cancer. When steroids upstream of the CYP17 pathway, (deoxycorticosterone and corticosterone), were used as biomarkers to assess the dose-dependent degree of target inhibition, a maximum effect appeared to be reached at a dose of 750 mg. Doses of 1000 and 2000 mg did not raise the steroid biomarker levels further, thus suggesting 750 mg was close to the optimal biological dose, and given the acceptable safety profile, the 1000 mg dose was selected both for the registration trial and as the label dose [44].

The proteasome inhibitor carfilzomib is used to treat multiple myeloma with a recommended label Cycle 1 dose of 20 mg/m^2^/day, and if tolerated, increasing the doses for subsequent cycles to 27 mg/m^2^/day. During clinical development, the Phase 1 trial assessed the inhibition of the chymotrypsin-like activity of the proteasome as a pharmacodynamic marker after single and multiple doses. Dose-dependent proteasome inhibition was observed in whole blood and peripheral blood mononuclear cells and appeared to plateau at doses of 11 mg/m^2^, which provided approximately 75 and 90 % proteasome inhibition following single and multiple doses, respectively [45]. The rationale for the selected dose of 20/27 mg/m^2^ was primarily based on the safety and clinical best ORR of 50 % in the Phase 1 dose escalation study and with the aim of providing a dose higher than the biologically active level of 11 mg/m^2^. The FDA approved the dose based on the overall clinical risk/benefit observed in the Phase 2 registration study.

## Summary of methods for E–R analysis applied in the evaluation of NME applications

This section categorizes and discusses the typical E–R analysis methods discussed in the “clinical pharmacology and biopharmaceutics reviews” for the oncology drugs approved during the survey period. E–R analysis may be further subcategorized into E–S and E–E analyses, which taken together provide an overall assessment for the “therapeutic index or therapeutic window” of the drug, and for “dose justification” or the need for “dose optimization” activities.

**Fig. 4:**
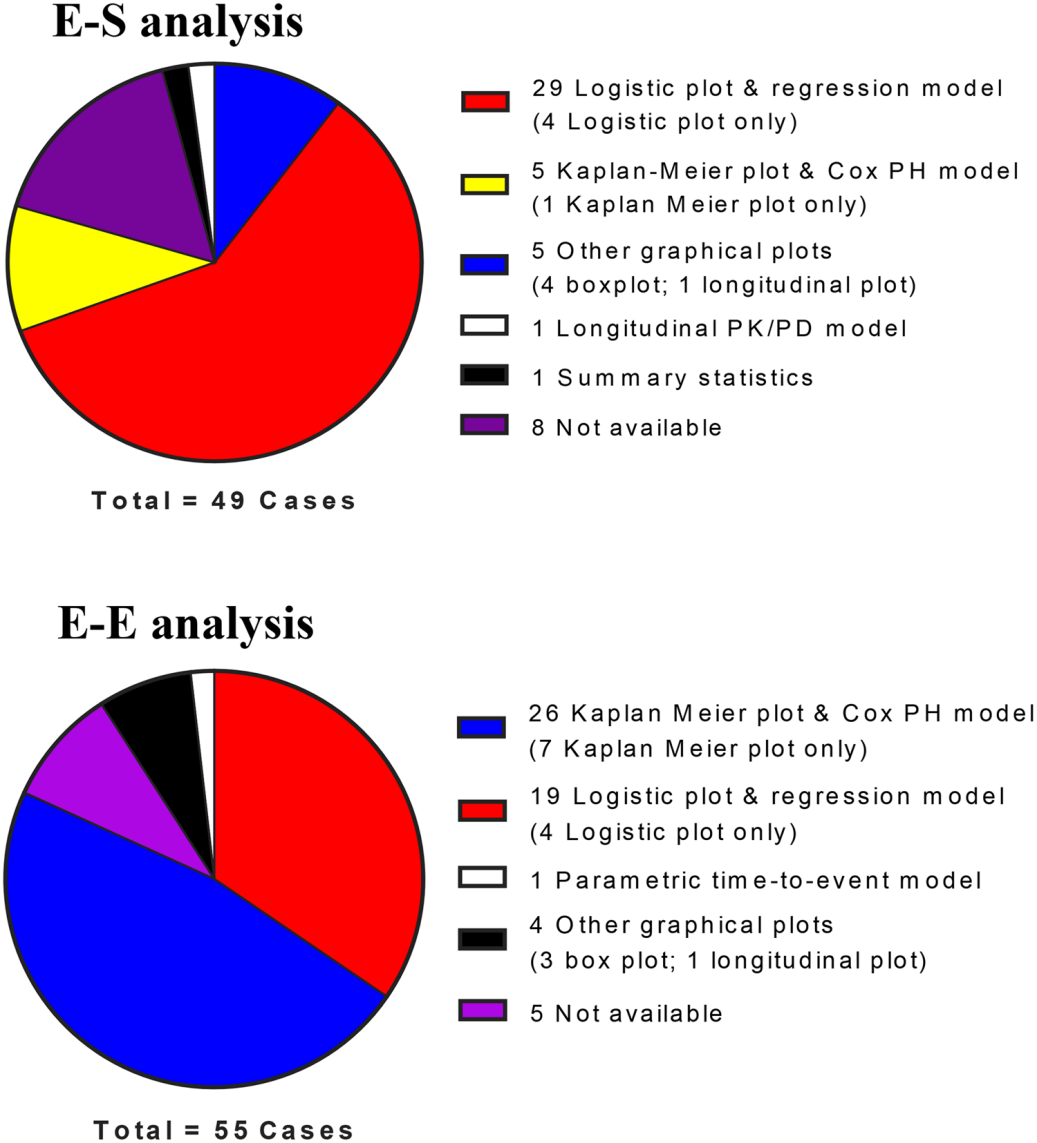
Summary of analysis techniques for the 41 drugs reviewed. **a** E–S analysis, **b** E–E analysis

Exposure–response analyses of some type (E–E, E–S, or both) were performed in 36 (88 %) of the 41 applications by either the sponsor or the FDA or both (Table 1). Figure 4 provides a summary of the E–R analysis methods for the 41 drugs reviewed. FDA methods were chosen to report when different from sponsors. Methods from sponsors were also included if the FDA did not conduct the analysis but agreed with the interpretation of the E–R relationship by the sponsor. Multiple methods could thus be included for a given drug. If the same method (e.g., logistic regression) was used for multiple endpoints in E–S (or E–E) analysis, it was only counted once. Only methods confirmed by FDA reviewers to be relevant to the clinical endpoints and dose justification were included, i.e. methods for certain exploratory analyses were omitted for lack of correlation to clinical endpoints. In total, for the 41 drugs, there were 49 E–S and 55 E–E analyses included.

### E–E analysis

**Fig. 5:**
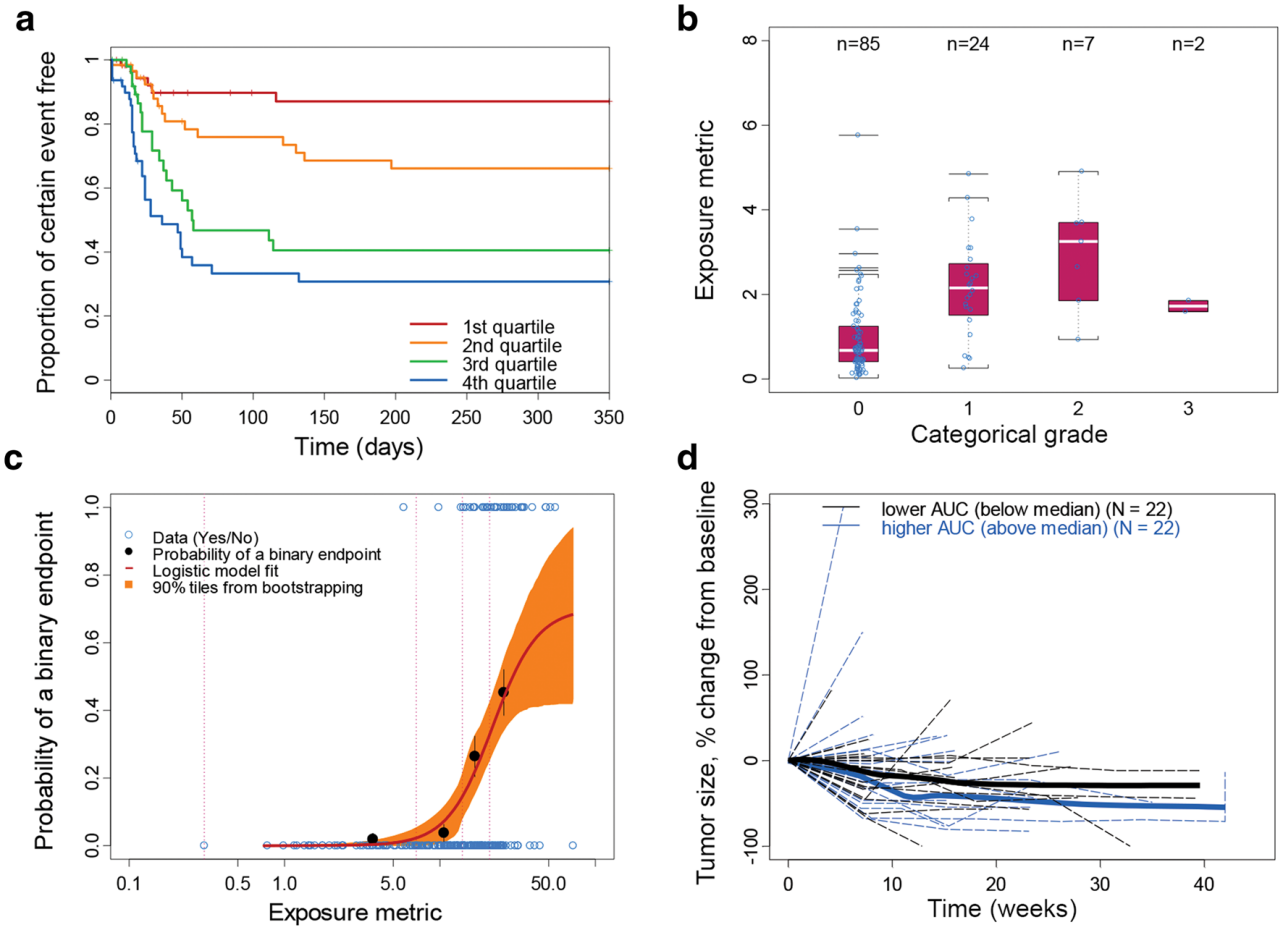
Representative plots for **a** Kaplan–Meier plot, **b** box plot, **c** logistic plot and regression, and **d** longitudinal plots for efficacy or safety endpoints

The aim of an E–E analysis is to assess the relationship between efficacy endpoints and PK exposure. The typical E–E analysis methods included are logistic regression and/or regression modeling (19 instances) for ORR or other binary type data (e.g., major cytogenetic responses, major hematologic response, pathologic complete response); and Kaplan–Meier plots and Cox proportional hazard (Cox PH) model for survival data (26 instances). Box plots and longitudinal plots as exploratory assessments are also used but to a lesser extent (3 and 1 instances, respectively). For example, the longitudinal tumor size time profile was assessed by category of AUC for vemurafenib [33]. Similar to the application of logistic regression for the occurrence of AEs, multivariate logistic regression could be used to examine the impact of PK exposure and baseline prognostic factors on the probability of binary efficacy endpoints. The Kaplan–Meier analyses of survival data stratified by PK exposure or dose is a qualitative way to explore the E–E relationship (Fig. 5a), as it does not account for the potential imbalances of risk factors between exposure or dose groups. There are only seven instances where Kaplan–Meier plots alone were used to inform an E–E relationship. In the majority of instances, Kaplan–Meier plots and the Cox PH model were used together in the E–E analysis. The Cox PH model can incorporate the impact of prognostic factors, and this may provide an estimated effect of exposure or dose on reducing the hazard rate, after adjusting for prognostic factors [25]. The Cox PH model assumed proportional hazard (time-constant hazard ratio, regardless of hazard rate fluctuations over time), given the linear relationship between natural logarithm of hazard function and the explanatory variables. Parametric time-to-event analysis, which considered the underlying hazard function, was also implemented for survival data (one instance: palbociclib). As compared to the Cox PH model, the parametric model can define the baseline hazard function, allow flexible covariate relationship to the hazard function, and can consider the time-varying covariates to better identify potential prognostic factors and the effect of treatment [56].

### E–S analysis

The aim of E–S analysis is to assess the tolerability (toxicity/safety) risk associated with variation of exposure. The grading of an AE is usually defined with specific methods, such as the Common Terminology Criteria for Adverse Events scoring system [57]. In the E–S analysis, safety endpoints may be expressed as categorical outcomes (e.g., Grade 0, 1, 2, 3, and 4 AE), binary outcomes, such as “0” for “non-severe AE” (e.g., Grade 0–2) and “1” for “severe AE” (e.g., Grade 3 and above), or “0” for “no AE related dose modification” and “1” for “AE related dose modification”, or continuous outcomes (e.g., neutrophil counts); exposure measures may include PK-related metrics (e.g., steady-state AUC, peak or trough concentration, either observed or population PK model predicted values), and dose-related metrics (e.g., dose intensity, BSA or BW, if BSA- or BW-based dosing), which can also be applied to E–E analysis. Graphical plots (e.g., box plot, logistic plot, and longitudinal plot) were the most common approaches for exploratory assessment of the E–S relationship. The box plot could be used to visually identify whether certain responses (categorical or binary endpoints) were associated with different exposures (representative plot is shown in Fig. 5b); however, these might fail to identify a relationship that is not sufficiently steep [58]. The box plot may also be confounded by a potential lack of balance of prognostic factors, since some patients may be predisposed to the safety risk because of their disease status. Thus, the box plot has not been widely used in the reviewed case examples (only in four instances).

Logistic plots and logistic regression models (Fig. 5c) are often used to explore and quantitate the E–S relationship. Compared to the box plot, the logistic plot is more sensitive for detection of the E–S relationship [58]. However, like with the box plot, conclusions may be influenced by confounding factors. The multivariate logistic regression model can isolate the relationship between the exposure variable and binary outcomes from the impact of confounding factors (i.e., demographic and prognostic factors). The probability of an AE at a given exposure or dose can be predicted thereafter by model simulation.

Logistic plots and regression models played a central role in E–S analysis in several cases (29 instances). Another approach, although much less utilized for E–S analysis (5 instances), is the Kaplan–Meier plot stratifying by exposure, with the time-to-event endpoints including time to certain “severe AE” occurrence, or time to first dose modification due to AE. The Kaplan–Meier plot can provide important information relating to whether higher PK exposure will be associated with a “time-related” temporal occurrence of events (Fig. 5a), which cannot be provided by logistic regression. In a situation where safety endpoints are expressed as continuous outcomes, such as in two cases of neutrophil counts over time, for obinutuzumab and palbociclib, the longitudinal plot (Fig. 5d) can be applied and stratified by obinutuzumab exposure groups to assess the impact on the endpoint time profile. As a more mechanistic approach, the longitudinal PK/PD model was implemented for palbociclib to evaluate the impact of dose and regimen on the neutrophil counts time profile.

Eight drugs did not have any E–S analysis, with seven out of eight having no or very limited PK. E–E analysis was able to be conducted for two out of those seven drugs (omacetaxine, mepesuccinate, and pertuzumab), using BSA or BW as the surrogate of exposure, which led to five instances of no E–E analysis conducted.

While E–R analyses are a common and critical element in dose evaluation in the SBA, we found that there were no instances where E–R analyses results were included in the product labeling for efficacy or safety endpoints. However, E–R language was common in the assessment of concentration-QT (electrocardiogram) effect.

Some more advanced pharmacometric methods have also been explored for the E–R analysis and regimen selection based on literature reports and our own experiences. However, these methods were not used for any of the 41 drugs reviewed here. These include, for example, longitudinal and repeat time-to-event model for categorical data for E–S [59, 60], and the use of case match analysis in the E–E modeling of survival data [61]. These methodologies may be more robust to refine E–R relationships by integrating data in a longitudinal fashion and balancing prognostic factors; however, comparisons of these methodologies for E–R assessment in oncology need further evaluation.

## Discussion and conclusion

Dose selection in oncology is complicated by a myriad of factors; several are exacerbated by the need to quickly deliver new effective therapies to cancer patients, who often have limited treatment options. Emerging concepts and strategies to determine the optimal dose in an era of molecularly targeted agents have been comprehensively reviewed by others [2, 3].

To extend the knowledge of dose selection and dose optimization during clinical drug development, we conducted a comprehensive survey of the label dose for the 41 NME applications for oncology drugs approved by the FDA from 2010 to the first quarter 2015. The public access to NME NDA/BLA applications and FDA reviews provided the ability to evaluate the strategies for dose selection of a drug during clinical development and the ability to identify the optimal dose. There were four key findings in our survey as follows: (1) Dose optimization-related PMRs/PMCs for NME applications are common and independent of drug class with approximately 27 % of drugs approved having postmarketing activities issued related to dose justification/optimization, including clinical trials to study alternative doses to the label dose, or to conduct further E–R analysis to justify the label dose. (2) Drug development with doses at the MTD, when identified while still common did not lead to an optimal dose, with nine of 21 (43 %) of drugs developed at the MTD having a PMR/PMC issued for dose optimization activities, which included five with trials to study a lower dose and four instances of additional E–R analyses. (3) Of 15 drugs where the MTD was not identified, the label dose was justified in 13 (87 %) with E–R analysis and two drugs having a PMR for trials at a higher dose. Thus, suggesting that the larger therapeutic window would allow studying if a higher dose would provide additional clinical benefit as suggested by E–E analysis. (4) The majority of KIs had an identified MTD and of 11 undergoing development at the MTD, four had PMR/PMCs for trials at a lower dose for safety concerns, and three for additional E–R analyses in ongoing trials, suggesting the relatively narrow therapeutic window of most KIs does not have an optimal/justified dose in majority of the cases when the MTD is used.

Of the drug approvals reviewed in our survey, two included upward dose titration during clinical development. Axitinib may be considered a prototypical example of success for this approach, which utilized stepwise upward dose titration across three doses in individual patients showing decreased PK variability and improved efficacy on retrospective analysis. However, less favorable study outcomes were noted in the case of afatinib, where intra-patient dose escalation from a lower dose to the established MTD in the registration trial resulted in less efficacy and increased toxicity. Dose titration as a means of decreasing PK variability and enhancing clinical benefit merits more exploration in future clinical trials, particularly when high PK variability is observed.

Currently, the application of blood biomarker data for dose selection is limited. Our survey indicates that overall, the use of biomarker data may be supportive in select cases for justification of the optimal dose in early clinical trials as well as for label dose justification, as illustrated by the recent drug approvals for enzalutamide and abiraterone for treating CRPC, ibrutinib for treating MCL and CLL, and carfilzomib approval for treating multiple myeloma.

The results of this survey show that E–E and/or E–S analyses have been consistently and widely applied by sponsors and the FDA, (approximately 90 % of reviews) for assessing the appropriateness of the proposed dose. Generally, if there is a trend toward greater efficacy related to increased exposure without compromising safety, a PMR/PMC may be issued to evaluate a higher dose, with the aim of improving patient outcomes. If there is both uncompromised efficacy and a trend toward improved safety profile with decreased exposure, a PMR/PMC may be issued to evaluate a lower dose.

In conclusion, the findings from our review support the need to develop clinical trial designs and data elements necessary to determine the optimal dose across all phases of drug development and also to fully assess the clinical potential of the NME [2–4]. There are multiple ongoing dose optimization PMR/PMC clinical trials and E–R analyses [9–12], and the results of these studies will provide valuable information in support of future strategic directions in oncology drug development and dose optimization.

## Electronic supplementary material

Supplementary material 1 (DOCX 13 kb)
