# Supplementary material for: A survey of new oncology drug approvals in the USA from 2010 to 2015: a focus on optimal dose and related postmarketing activities
Source: Cancer Chemother Pharmacol. 2016 Jan 25;77:459–76. doi: 10.1007/s00280-015-2931-4 (PMC4767861; doi:10.1007/s00280-015-2931-4)
Supplement: Supplementary file 1 — Supplementary material 1 (DOCX 13 kb) [file 280_2015_2931_MOESM1_ESM.docx]

**Supplemental information**

**Supplemental Table 1**: Summary of 41 new molecular entities (NMEs) approved for cancer treatment from 2010 through the first quarter of 2015

|  |  | **Number of drugs** | **Dose optimization related PMR/PMCs** | | | |
| --- | --- | --- | --- | --- | --- | --- |
|  |  |  | Total | Trial testing a higher dose | Trial testing a lower dose | Additional E-R analysis |
| LM | mAbs | 9 | 1 | 1  Ipilimumab | 0 | 0 |
|  | Enzyme | 1 | 0 | 0 | 0 | 0 |
|  | Fusion protein | 1 | 0 | 0 | 0 | 0 |
|  | ADCs | 2 | 1 | 0 | 0 | 1  Ado-trastuzumab emtansine |
| SM | KIs | 16 | 7 | 0 | 4  Vandetanib, Cabozantinib, Ceritinib, Lenvatinib | 3  Ponatinib,  Regorafenib,  Crizotinib |
|  | Other molecular targeted agents | 8 | 1 | 0 | 1 Panobinostat | 0 |
|  | Chemotherapeutic agents | 3 | 0 | 0 | 0 | 0 |
|  | Radioactive agents | 1 | 1 | 1  Radium-223 | 0 | 0 |
|  | Sum | **41** | **11** | 2 | 5 | 4 |

ADCs, antibody-drug conjugates; E-R, exposure-response; LM, large molecule; mAbs, monoclonal antibodies; PMC, postmarketing commitment; PMR, postmarketing requirement; SM, small molecule; KIs, tyrosine kinase inhibitors
